# Characterization and Bioactive Potential of Carotenoid Lutein from *Gordonia rubripertncta* GH-1 Isolated from Traditional Pixian Douban

**DOI:** 10.3390/foods11223649

**Published:** 2022-11-15

**Authors:** Qing Zhang, Jie Wang, Chanyuan Li, Miaoxin Zheng, Zihan He, Yuting Zou, Haibo Xiong, Bitao Xu, Wenliang Xiang, Jie Tang

**Affiliations:** Key Laboratory of Food Microbiology, School of Food and Bioengineering, Xihua University, Chengdu 610039, China

**Keywords:** carotenoid lutein, *Gordonia rubripertincta*, characterization, antioxidant, ocular apoptosis cells

## Abstract

The characterization and bioactive properties of carotenoid produced by *Gordonia rubripertincta* GH-1 originating from Pixian Douban (PXDB), the Chinese traditional condiment, was investigated. The produced and purified yellow pigment was characterized by ultraviolet-visible spectroscopy (UV-Vis), Fourier transformed infrared (FTIR), nuclear magnetic resonance (NMR), and high-resolution mass spectrometry (HRMS), and was identified as carotenoid lutein. Additionally, the bioactive activity of lutein from *G. rubripertincta* GH-1 was evaluated by measuring the free radical scavenging capacity in vitro and feeding zebrafish lutein through aqueous solution. The results showed that the carotenoid lutein had strong antioxidant capacity and a protective effect on zebrafish eye cells, which could inhibit the apoptosis of eye cells in a concentration dependent manner. The results suggested that carotenoid lutein from *G. rubripertincta* GH-1 could be utilized as a potential source of natural antioxidants or functional additives for food/pharmaceutical industries.

## 1. Introduction

Natural and synthetic pigments are widely used in foods, cosmetics, medicines, and in other products. Currently, there exists an increasing trend of public awareness on adopting measures that are environmentally friendly and that improve human safety [1]. People have shown their aversion to synthetic pigments, therefore natural pigments are preferred by consumers [2].

Carotenoids are the most widespread group of natural pigments [3]. The color of carotenoids ranges from yellow to orange-red. Carotenoids naturally exist in photosynthetic organisms as a beneficial fat-soluble pigment [4,5]. Furthermore, natural carotenoids are also found in certain non-photosynthetic organisms where they protect cells from light and oxygen damage [6]. In addition, carotenoids show antioxidant, antitumor, and antimicrobial properties; therefore, they are widely used in foods, medicines, feeds, and cosmetics fields [6,7]. Currently, natural carotenoids are mainly produced by plants; however, the natural carotenoid production through extraction from plants is limited by uncertain seasonal and geographic variability [8]. This leads to research of microbial fermentation for the production of natural carotenoids as potential alternatives.

Bacterial production of carotenoids have recently come into the spotlight due to their various advantages, such as fast and easy cultivation, higher biomass in shorter periods of time, no seasonal restrictions, nontoxicity, and efficient extraction process [6]. Bacteria species such as *Micrococcus* spp., *Flavobacterium* spp., *Agrobacterium* spp., *Chromobacterium* spp., *Arthrobacter* spp., *Serratia marcescens*, *Pseudomonas aeruginosa*, *Rheinheimera* spp., *Gordonia alkanivorans*, *Gordonia terrae* and *Brevibacterium linens* [6,9,10,11,12] have been reported for carotenoid production, and studies demonstrate that commercial microbial production of carotenoid is still in the research and development phase. Currently, the growing worldwide interest in the development and production of carotenoids from natural sources has boosted researchers’ interest in finding new microorganisms for naturally derived carotenoid from different sources in the environment.

Pixian Douban (PXDB), a historical traditional semi-solid condiment from southwestern China, which naturally ferments with koji (usually inoculated with *Aspergillus oryzae*) as a starter, has unique ingredients, including broad bean, red pepper, wheat flour, and salt, and has a bright red color [13]. Diverse microorganisms exist during natural fermentation, playing a significant role in the quality of PXDB [14]. However, carotenoid pigments generated by specific microorganisms present in PXDB, and which may contributing to PXDB coloration, have not yet been studied. It was previously reported that the pigments produced on the surface of smear-ripened cheese benefit from the production of microorganisms [15,16]. Therefore, seeking carotenoid pigment producing strains from PXDB is essential.

Carotenoids have gained a reputation not only as nutraceuticals and nutricosmetics, but also as biologically active molecules [6]. Most of studies on carotenoids are based on their robust antioxidant capabilities [1,3]. Currently, there has been a growing interest in the study of carotenoid’s (especially lutein’s) influence on eye problems, such as cataract and age-related macular degeneration, as well as defense of the retina against exposure to phototoxic light damages [17,18,19,20]. To better understand the eye cell protective properties of extracted carotenoid from *G. rubripertncta*, a zebrafish model was used to evaluate the visual protective activities. The zebrafish has become an ideal biological model to study human diseases because of its short growth cycle, transparent embryonic development, and highly similar eye structure to human beings [21,22]. To the best of our knowledge, this is the first work that considers the bioactivities of carotenoid lutein from *G. rubripertncta* in preventing eye cell apoptosis of zebrafish model.

In this work, a novel strain with pigment biosynthesis ability was isolated and identified as *Gordonia rubripertncta*. The structural characteristics of the extracted pigment were explored by ultraviolet-visible spectroscopy (UV-Vis), Fourier transformed infrared (FTIR), nuclear magnetic resonance (NMR), and high-resolution mass spectrometry (HRMS), and characterized as lutein. In addition, three kinds of free radical scavenging capacities were measured to evaluate the in vitro antioxidant capacity of carotenoid lutein. Moreover, the protective effect of lutein on vision problems has been determined using the zebrafish model. These findings seek to provide a comprehensive understanding of natural biomolecules of carotenoid lutein produced by *G. rubripertncta* GH-1 from traditional PXDB.

## 2. Materials and Methods

### 2.1. Strain, Culture Conditions, and Identification

Pigmented colonies of potential carotenoid producers were screened and isolated from PXDB, which was obtained from a traditional PXDB brewing company in Sichuan province of China. Samples of PXDB (2 g) were aseptically weighted and homogenized into 100 mL of selection medium (5 g/L acid hydrolyzed cassin, 10 g/L yeast extract, 5 g/L bacterial peptone, 3 g/L trisodium citrate, 3 g/L potassium chloride, 18 g/L magnesium sulfate, 0.2 g/L calcium chloride, and 0.001 g/L ferrous sulfate), and cultured at 30 °C under light for 5 d. The cultured samples were then serially diluted and 0.1 mL of dilution was spread on a selection medium agar plate and cultured at 30 °C under light for 72 h. An orange colored colony was isolated and named as strain GH-1 for further analysis. The strain GH-1 was cultured in medium (20 g/L sucrose, 4 g/L potassium nitrate, 0.4 g/L magnesium sulphate, 0.015 g/L ferrous sulfates, 2 g/L potassium dihydrogen phosphate, 3 g/L dibasic sodium phosphate, and 2 g/L trisodium citrate dihydrate) at 30 °C, 150 rpm for 48 h for subsequent identification. The colony and cell morphology of strain GH-1 were observed by plate streaking and scanning electron microscopy (Inspect F50, FEI, Hillsboro, OR, USA), respectively. The 16S rRNA sequence was used to analyze the molecular biology of the strain, and a phylogenetic tree was constructed and analyzed with the neighbor-joining method using the MEGA software, version 5.0.

### 2.2. Preparation and Purification of the Pigment

#### 2.2.1. Preparation of Crude Pigment

Pigments were extracted as described by Hu, et al. [23] with some modifications. The extracted cells were obtained from a 5-d culture of strain GH-1 in medium (30 °C under light, 150 rpm). The cells were then centrifuged at 8000× *g* force for 10 min at 4 °C using a tabletop ultracentrifuge (Optima Max-XP, Beckman Coulter, Brea, CA, USA) and the supernatant was discarded, followed by washing cells with sterile saline thrice. Next, the collected cells were homogenized in acetone by vortexing, and the mixture was disrupted using an ultrasonic homogenizer (Scientz-IID, Ningbo, Zhejiang, China) in an ice bath. Next, the supernatant was collected by centrifuging the pigment extract at 8000× *g* force for 10 min at 4 °C. The resulting extract was concentrated at 40 °C with a rotary evaporator (RE-52AA, Shanghai Yarong Biochemistry Instrument Corporation, Shanghai, China), and the crude pigment extract was stored at −50 °C until use.

#### 2.2.2. Purification by Silica Gel Column Chromatography

Silica gel column chromatography was used to purify the crude pigment. Silica gel (200–300 mesh) was activated at 110 °C for 2 h to remove moisture using a wet packing column (30 mm × 300 mm). The column was equilibrated with petroleum ether and acetone (1:4, *v*/*v*). Prior to sample separation, the level of liquid was lowered equal to the stationary phase. The crude pigment obtained was loaded into the column and the elution was performed with a gradient of petroleum ether and acetone at a constant flow rate of 15 mL min^−1^. The eluted fractions were screened using thin-layer silica gel chromatography plates (50 mm × 100 mm, GF254) with petroleum ether and acetone (1:4, *v*/*v*) as developing reagents. The fractions collected were analyzed by a high-performance liquid chromatography (HPLC) instrument (Waters Alliance 2695, Waters Corp., Milford, MA, USA) equipped with a UV-visible detector. Analyses were performed on an Agilent Eclipse Plus C18 column (4.6 mm × 150 mm, 5 μm) at 30 °C. The mobile phases used were methanol (A) and H_2_O (B) with a gradient elution mode as follows: 90% A at 0–8 min, 100% A at 8–21 min, 90% A at 21–30 min. The flow rate of the mobile phase was 1 mL/min. The eluate was monitored at 473 nm. Based on the peak analysis of HPLC, the purified components were collected, dried at 40 °C, and dissolved in methanol. Before carotenoids analysis, the purified pigment was filtered through a filter (0.22 μm).

### 2.3. Characterization of Pigment Structure

#### 2.3.1. UV and Infrared Spectra of Pigment

Visible light absorption spectra of the carotenoids were detected using a UV spectrophotometer 2400 (Shanghai Sunny Hengping Scientific Instrument Co., Ltd., Shanghai, China) at wavelengths of 350–600 nm [24]. Fourier transform infrared spectroscopy (FT-IR) (L1,600,400 Spectrum Two, PerkinElmer, MA, USA) was used to identify the main functional groups of the purified carotenoids using the attenuated total reflection (ATR) method in the wavelength range of 4000–450 cm^−1^ [25].

#### 2.3.2. Nuclear Magnetic Resonance (NMR) Analysis

The purified pigment (30 mg) was dissolved in dimethyl sulfoxide (DMSO) and transferred into a nuclear magnet tube in the dark. The 1H and 13C NMR spectra were acquired using a Bruker Avance 400 and a Bruker DPX 300 spectrometer, respectively, at a sample temperature of 25 °C.

#### 2.3.3. Mass Spectrometry Analysis UV and Infrared Spectra of Pigment

MicrOTOF-QII mass spectrometry (Bruker, Bremen, Germany) and AB SCIEX (X500R, Framingham, MA, USA) mass spectrometry was used for pigment identification. The electrospray ionization-time of flight-mass spectrum (ESI-TOF-MS) spectra were obtained by scanning from *m/z* 50 to 1200 with a dry heater at 180 °C, capillary voltage of 4500 V, and collision cell RF of 150.0 Vpp [26]. To acquire the specific structure of the pigment produced by strain GH-1, high-resolution mass spectrometry (HRMS) was used to analyze the ion fragments of the pigment [27]. The analysis was conducted using an Ultimate UHPLC LP-C18 column (2.1 mm × 100 mm, 1.8 μm, Welch, Shanghai, China). The gradient program used is listed in supplementary materials Table S1. The gradient elution program was performed using two solvents: solvent A (H_2_O with 0.1% formic acid) and solvent B (acetonitrile) at a flow rate of 0.1 mL/min. The Mass Spectrometry/Mass Spectrometry (MS/MS) spectra was obtained by scanning from *m/z* 100 to 600 with a capillary voltage of 5500 V, declustering potential of 70 V, and ion source temperature of 400 °C (ESI+).

### 2.4. Bioactivities of Carotenoid Lutein

#### 2.4.1. Assay of Antioxidant Activities

In vitro antioxidant activities of purified carotenoid lutein was evaluated by determining its free radical scavenging ability. TDPPH, hydroxyl and superoxide free radicals scavenging activity were measured in terms of the method describe by Zhang, et al. [28].

#### 2.4.2. Inhibit Zebrafish Eye Cells Apoptosis Activity

The study protocol was approved by the ethics review board of College of Food and Bioengineering of Xihua University. We have obtained written informed consent from all study participants. All of the procedures were performed in accordance with the Declaration of Helsinki and relevant policies in China. The zebrafish (*Danio rerio*) was used as a model to evaluate the eye cell apoptosis inhibition activity of lutein. Wild-type AB zebrafish at 1 day post fertilization (dpf) were randomly selected in 6-well plates (Nest Biotech, China), with 30 fishes per well (experimental group). Zebrafish were fed with lutein in the form of aqueous solution (0.049, 0.098, 0.195, 0.390, 0.780, 1.560 μg/mL). The volume of each well is 3 mL. After 24 h treatment at 28 °C, the maximum tolerance concentration (MTC) of lutein on model zebrafish was determined.

Then, the zebrafish eye cells apoptosis inhibition activity of lutein was analyzed. Zebrafish were fed with lutein in the form of aqueous solution (0.195, 0.390, 0.780 μg/mL), and the L-reduced glutathione (GSH, 615 μg/mL) was the positive control group (SLCF2362, Sigma). In addition, the normal control group and model control group were set at the same time. Except the normal control group, the other experimental groups were given morpholine ethyl mycophenolate to establish zebrafish eye apoptosis model. Then acridine orange (AO) staining was performed after treatment at 28 °C for 24 h. After staining, 10 zebrafish in each experimental group were randomly selected and photographed under a fluorescence microscope (AZ100, Nikon, Tokyo, Japan). In addition, advanced image processing software (NIS-Elements D 3.20, Nikon) was used to analyze and collect data, analyze the fluorescence intensity of eye apoptotic cells, and evaluate the eye protection effect of lutein produced by strain GH-1 according to the statistical analysis results of this index.

### 2.5. Statistical Analysis

The statistical results were expressed by mean ± SE. In addition, the SPSS 26.0 software was used for statistical analysis, *p* < 0.05 was considered statistically significant.

## 3. Results and Discussion

### 3.1. Identification of GH-1 Strain

**Figure 1:**
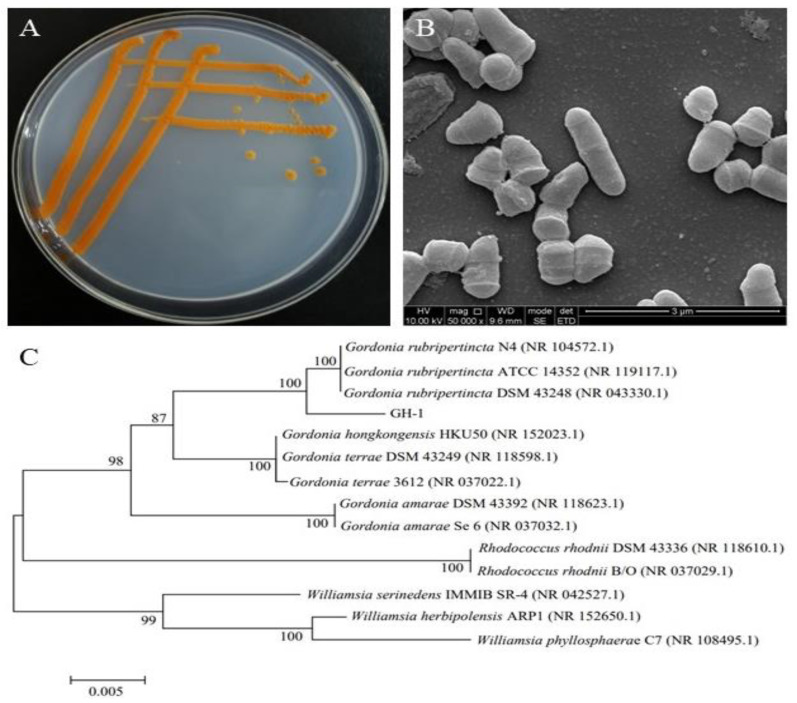
Colony (**A**), cell morphological (**B**) characteristics and phylogenetic tree (**C**) of strain GH-1.

A yellow pigment-producing strain, GH-1, was screened from PXDB samples and was cultured in medium agar at 30 °C under light for 72 h. The colony and morphological characteristics of isolated GH-1 are shown in Figure 1. The strain colony morphology (Figure 1A) shows round, opaque, raised, and wrinkled orange colonies on the plate. Under a scanning electron microscope, the cells of GH-1 were 1.5–2.0 μm in length and 0.3–0.5 μm in width, and rodding in shape without spores (Figure 1B). The taxonomic and phylogenetic relationships of GH-1, 16S rRNA gene sequencing were used for molecular identification of the strain. The 16S rRNA sequence of strain GH-1 showed 99% similarity to *Gordonia rubripertncta* (Figure 1C). Combined morphological and 16S rRNA gene phylogeny analysis of GH-1 was related to *G. rubripertncta*. (Collection number: CGMCC 21239; GenBank accession number: MW280135).

### 3.2. UV and FT-IR Spectral Analysis of Purified Carotenoid

The *G. rubripertncta* GH-1 produced the yellow pigment yield of 7.24 mg/L. The HPLC result of the extracted and purified pigment from silica gel column chromatography was showed in Supplementary Materials Figure S1. Then the purified pigment was investigated for its structure characterization and bioactivity potential.

**Figure 2:**
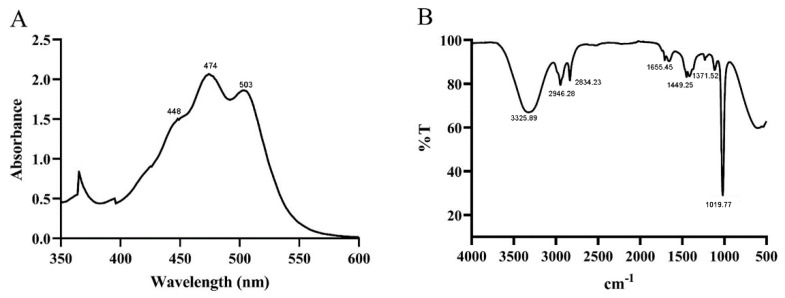
UV-Vis (**A**) and FT-IR (**B**) spectrum of the purified pigment from *G. rubripertincta* GH-1.

The ability to absorb visible light (400–500 nm) enables carotenoid to play a role in light quenching and protecting cells from light damage, and assists in carotenoid identification [29]. The UV absorption spectrum of the purified pigments showed maximum absorption at 448, 474, and 503 nm, respectively (Figure 2A). Furthermore, it also exhibited the typical three-fingered peak, a common characteristic of carotenoids [30]. Thus, the results indicated that the pigment produced by *G. rubripertncta* GH-1 might belong to the carotenoid family [31,32].

To characterize the functional group information of the carotenoid, FT-IR spectra was used to record an absorbance range of 4000–450 cm^−1^ (Figure 2B). The purified pigment showed a broad absorption peak at approximately 3325 cm^−1^, corresponding to the intense hydroxyl group (-OH) stretching vibration [33]. In addition, two absorption peaks appeared ranging between 2946 cm^−1^ and 2834 cm^−1^, due to the sp3 alkyl C-H group [25], and the weak peak at approximately 1655 cm^−1^ due to the C=C bond. Furthermore, the asymmetrical and symmetrical vibrations (-CH groups) were observed at 1449 and 1371 cm^−1^, respectively [34], and the strong absorption peak at 1019 cm^−1^ was assigned to the C-O stretching vibration [35,36]. The results indicated that the pigment was an oxygen-containing lutein substance.

### 3.3. NMR Spectroscopy Analysis of Purified Carotenoid

**Figure 3:**
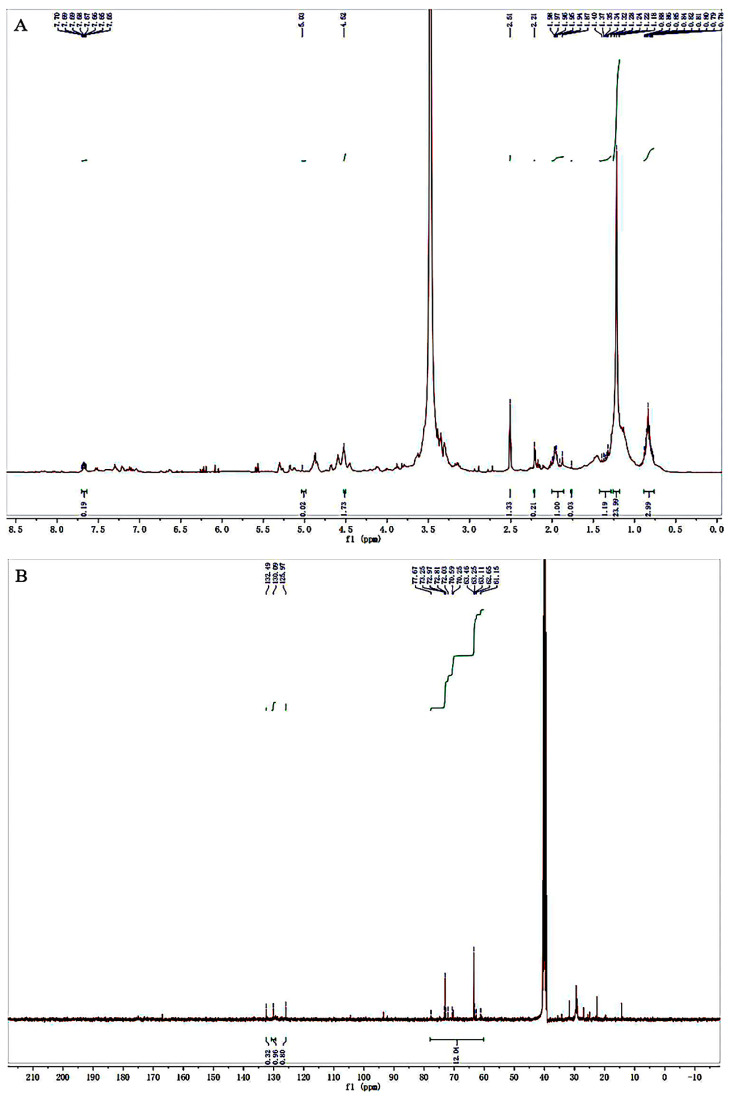
^1^H NMR (**A**) and ^13^C NMR (**B**) spectra of purified pigment from *G. rubripertincta* GH-1.

The ^1^H and ^13^C NMR spectra were used to analyze the structural characteristics of the purified pigment. In the ^1^H NMR spectrum (Figure 3A), multiple peaks appeared at δ 0.77–2.0 ppm, characteristic peaks of -CH_3_, -CH_2_, and -CH in the pigment. In addition, an absorption peak obtained at approximately δ 2.21 was attributed to the methylene protons on the heterocyclic ring [37]. The peak at δ 2.51 ppm was attributed to dimethyl sulfoxide, and the strong peak around δ 3.31 ppm was attributed to the water in the solution. Furthermore, a strong signal was observed at δ 4.52 ppm due to the hydroxyl group on the structure, and other multiple peaks that appeared at δ 5.03–7.70 ppm corresponded to the vinyl proton [38], which was consistent with the FT-IR analysis results.

As shown in the ^13^C NMR spectrum (Figure 3B), the peaks appearing at around δ 61.15–77.67 ppm were attributed to the carbon connected to the hydroxyl group. The olefin carbon peaks of the pigment were concentrated in the δ 125.97–132.49 ppm range [38].

### 3.4. Mass Spectrometry Analysis of Purified Carotenoid

**Figure 4:**
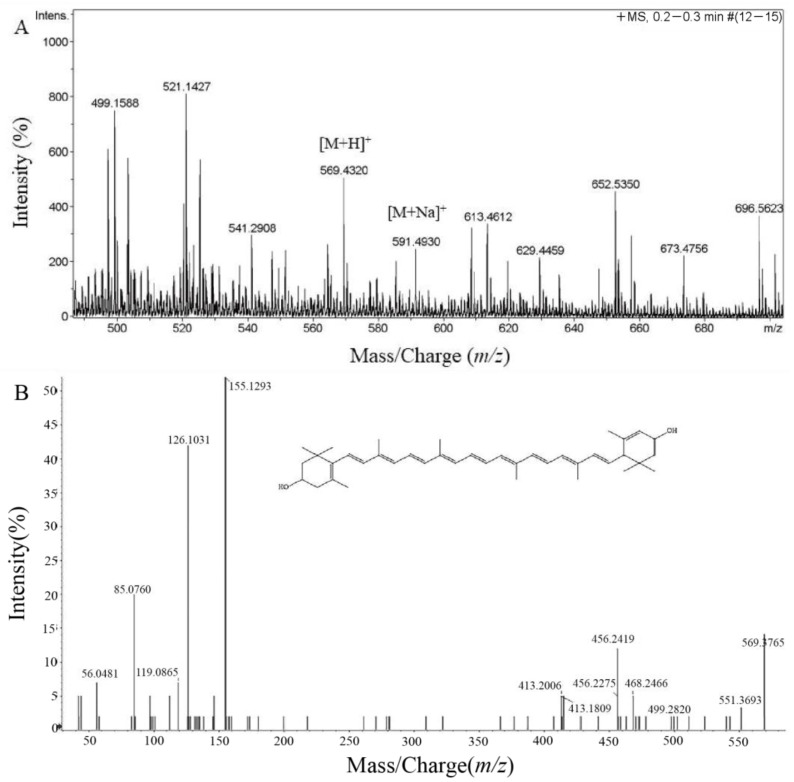
MS (**A**) and MS/MS (**B**) spectrum of purified pigment from *G. rubripertincta* GH-1.

Further identification of the primary carotenoid was conducted using HRMS. As shown in Figure 4A, a molecular ion peak was observed at *m/z* 569.4320, corresponding to the [M+H]^+^ ion, indicating the molecular formula C_40_H_56_O_2_. In addition, a peak appeared at *m/z* 591.4930, which corresponds to the sodium salt adducts [M+Na]^+^ ion [38]. To obtain the specific structure of the carotenoid, the (MS/MS) spectra were analyzed. The MS/MS spectrum in Figure 4B shows that a fragment ion at *m/z* 551.3693 was found, corresponding to [M-H_2_O]^+^ ion [39]. Furthermore, characteristic fragment ions were observed, such as *m/z* 499.2820, *m/z* 413.2009, and *m/z* 155.1293. Therefore, we inferred that the carotenoid was lutein and chemical structure was showed in Figure 4B. Similar to other carotenoids, the structure of the lutein molecule contains a backbone of conjugated double carbon-carbon bonds. Notably, the two hydroxyl groups on both ends of the lutein molecule differentiates it from zeaxanthin and other carotenoids [18]. Green plants are rich in lutein. However, because the hydroxyl groups in lutein, it can be esterified with fatty acids and form mono- and diacylated derivatives in plant cells [40]. The lutein extracted from marigold flowers is mostly found in its esterified form, and it needs to be further purified with solvents to its free lutein form [41].

### 3.5. Antioxidant Activity of Lutein

**Figure 5:**
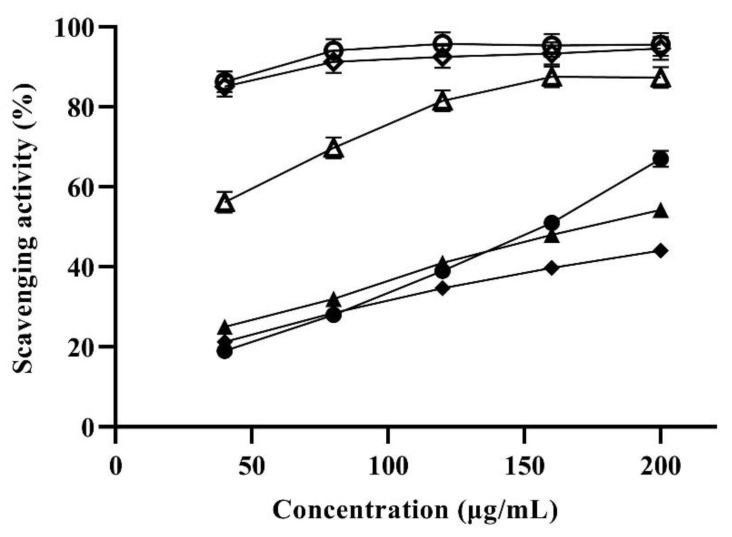
In vitro antioxidant activity of lutein from *G. rubripertincta* GH-1 using ascorbic acid (Vc) as reference. The ○, △ and ◇ indicate the DPPH radical, hydroxyl radical and superoxide radical scavenging activity of Vc reference, respectively. The ⚫, ▲ and ◆ indicate the DPPH radical, hydroxyl radical and superoxide radical scavenging activity of lutein, respectively.

Previous studies have shown that common human diseases such as cancer and tumors were closely related to free radicals [42]. Antioxidants such as carotenoids are considered to be effective in preventing the formation of radicals and scavenge reactive oxygen species [29]. Therefore, the antioxidant potential of lutein produced by strain GH-1 was evaluated according to the scavenging activities of three free radicals. The results indicated that the scavenging activities of lutein to DPPH, hydroxyl and superoxide free radicals were concentration dependent (Figure 5).

The scavenging activity of the carotenoid lutein sample for the DPPH radical was shown in Figure 5. Its scavenging activities enhanced with increasing concentration in the range of 0–200 μg/mL. When the concentration reached 200 μg/mL, the lutein and ascorbic acid (Vc) scavenging capacity reached maximum values of 67.0% and 95.6%, respectively. In addition, the scavenging ability of lutein sample against hydroxyl and superoxide radicals gradually increased with the concentration of lutein in the range of 0–200 μg/mL. At a concentration of 200 μg/mL, the lutein scavenging capacity reached a maximum value of 54.3% and 44.1%, respectively. It is worthy to note that the maximum scavenging capacity of lutein to hydroxyl radicals was close to that of Vc at 40 μg/mL. Studies have reported that carotenoids have strong antioxidant activity, which is due to the existence of conjugated double bonds in their chemical structure [43]. In addition, the two hydroxyl groups on both ends of the lutein are responsible for their elevated chemical reactivity with singlet oxygen [44]. The above results demonstrated that lutein produced by strain GH-1 can be used as potential antioxidants.

### 3.6. Inhibitory Effect of Lutein on Ocular Apoptotic Cells in Zebrafish

The eye structure of zebrafish is similar to humans and highly conservative, so it can be used as an ideal model for human research on eye diseases [45]. What is more, the zebrafish models are superior to other animal models in transparency, which is more conducive to observation in vitro [46]. Here, the efficacy of lutein produced by *G. rubripertncta* GH-1 in preventing eye cell apoptosis was evaluated by feeding the zebrafish with an aqueous solution. GSH is an important cellular antioxidant and maintains cellular redox homeostasis; it is regarded as a critical protective factor for cataracts, glaucoma therapy, and the lens epithelial cells, and inhibit the onset [47,48,49]. Therefore, in the present study, GSH was chosen as positive control.

The toxicity of lutein was assessed based on observed behavioral phenotypes of zebrafish. With the increasing of lutein concentration, the involuntary spasm of the zebrafish tail was observed. As shown in Table 1, after 24 h treatment, the behavior phenotypes of zebrafish in normal control group and model control group were not significantly abnormal. In addition, the phenotype of lutein concentration groups (0.049, 0.098, 0.195, 0.390, 0.780 μg/mL) were similar to that of the model control group, while the phenotype of 1.560 μg/mL concentration group was worse than that of the model control group. Therefore, we inferred that the MTC value of lutein on the inhibitory effect of zebrafish eye apoptotic cell was 0.780 μg/mL.

Previous studies have shown that high carotenoid lutein intake is beneficial to eye problems, such as age-related macular degeneration (AMD) and cataracts, whether through food or as a nutritional supplement [19]. The experimental results showed (Table 2) that the fluorescence intensity of zebrafish eye apoptotic cells in the model control group is significantly higher than that in the normal control group, indicating that the experimental model is reliable. In addition, compared with the model control group, the fluorescence intensity of zebrafish eye apoptotic cells in the positive control group (L-reduced glutathione) was 761,321 pixels. The difference was extra significant (*p* < 0.001), indicating that the follow-up experimental results were reliable.

**Figure 6:**
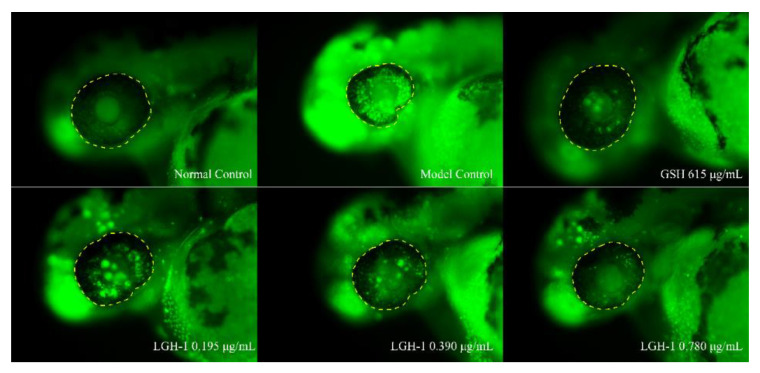
The typical phenotype of apoptotic cells in zebrafish eyes after lutein from *G. rubripertincta* GH-1 treatment. The yellow dotted line is the analysis site of zebrafish eyes, and the green fluorescent point is apoptotic cells.

**Figure 7:**
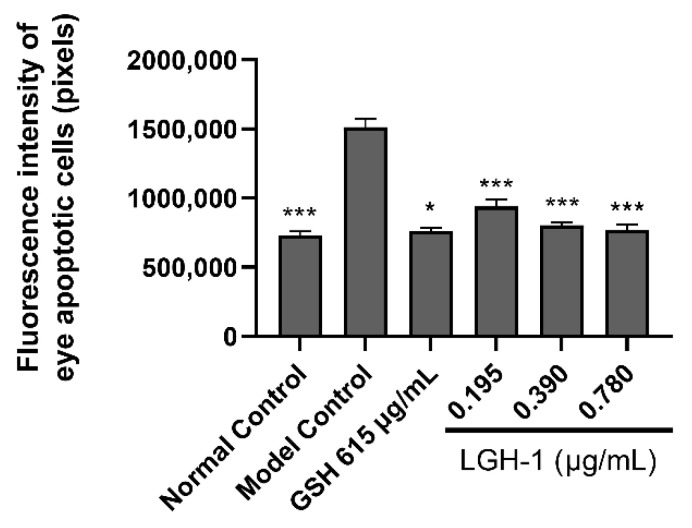
Fluorescence intensity of apoptotic cells in zebrafish eyes treated with lutein from *G. rubripertincta* GH-1 (* *p* < 0.05, *** *p* < 0.001).

The phenotype and fluorescence intensity of apoptotic cells in zebrafish eyes after lutein treatment were shown in Figure 6 and Figure 7, respectively. Compared with the model control group, the fluorescence intensities of apoptotic cells in zebrafish eyes at low (0.195 μg/mL), medium (0.390 μg/mL) and high (0.780 μg/mL) concentrations of lutein were 940,424 pixels (*p* < 0.05), 804,441 pixels (*p* < 0.001), 768,614 pixels (*p* < 0.001), respectively. In addition, our results indicated that model control group ocular cells emitted a bright green fluorescence. Meanwhile, the fluorescence intensity of zebrafish eye cells decreased significantly after fed lutein. It is worth noting that the fluorescence intensity of zebrafish eye cells was basically the same as that of the positive control group (L-reduced glutathione, 615 μg/mL) after feeding the zebrafish at high concentration (0.780 μg/mL), indicating that the lutein produced by strain GH-1 can effectively inhibit the apoptosis of zebrafish eye cells. In addition, the results of Saidi, et al. [45] reported that the injection of pure zeaxanthin into zebrafish eyes could improve visual performance. The lutein and zeaxanthin are isomers, and combined with the above research results, we inferred that the carotenoid lutein produced by strain GH-1 had protective effect on zebrafish eyes, which was manifested in improving eye cell apoptosis in a concentration dependent manner. Therefore, the carotenoid lutein produced by strain GH-1 can be used as a potential natural agent for the treatment of eye diseases.

## 4. Conclusions

The study identified a yellow pigment-producing strain GH-1 isolated from PXDB as *Gordonia rubripertncata*. Structural characterization of the pigment extracted from *G. rubripertncta* GH-1 was conducted by UV-Vis, FTIR, NMR, and HRMS methodologies, and the pigment was characterized as carotenoid lutein. The carotenoid lutein exhibited excellent antioxidant activity in vitro and protective bioactivity potential in apoptotic cells of zebrafish eyes. Collectively, the findings indicate that the carotenoid lutein from *G. rubripertncta* GH-1 possesses multiple biological activities, and may potentially be explored for food/pharmaceutical industrial application.

## Figures and Tables

**Table 1:** MTC results of different concentrations of lutein (n = 30).

Group	Concentration (μg/mL)	Mortality (%)	Phenotype
Normal Control	-	0	No obvious abnormality No obvious abnormality
Model Control	-	0	
LGH-1	0.049	0	The state was similar to that of the model control
	0.098	0	
	0.195	0	
	0.390	0	
	0.780	0	
	1.560	0	The state was worse than that of the model control

Note: LGH-1 refer to the lutein produced by *G. rubripertncta* GH-1.

**Table 2:** Evaluation results of lutein eye protection efficacy (*n* = 10).

Group	Concentration (μg/mL)	Fluorescence Intensity of Ocular Apoptotic Cells (Pixel, Mean ± SE)
Normal Control	-	733,008 ± 27,451 ***
Model Control	-	1,513,891 ± 58,443
GSH	615	761,321 ± 23,260 ***
LGH-1	0.195	940,424 ± 51,050 *
	0.390	804,441 ± 17,855 ***
	0.780	768,614 ± 40,160 ***

Note: compared with the model control group, * *p* < 0.05, *** *p* < 0.001.

## Data Availability

Data is contained within the article or Supplementary Material.

## Supplementary Materials

The following are available online at https://www.mdpi.com/article/10.3390/foods11223649/s1, Table S1: Elution procedure for high resolution mass spectrometry. Figure S1: HPLC chromatogram of the purified pigment from silica gel column chromatography.
