# Supplementary material for: Characterization and Bioactive Potential of Carotenoid Lutein from Gordonia rubripertncta GH-1 Isolated from Traditional Pixian Douban
Source: Foods. 2022 Nov 15;11(22):3649. doi: 10.3390/foods11223649 (PMC9689138; doi:10.3390/foods11223649)
Supplement: Supplementary file 1 [file foods-11-03649-s001.zip › foods-1965786-supplementary.pdf]

Table S1. Elution procedure for high resolution mass spectrometry.

Time (min)	Flow rate (mL/min)	Solvent A	Solvent B
0	0.1	5	95
1	0.1	5	95
4	0.1	80	20
11.38	0.1	100	0
15	0.1	100	0
18	0.1	5	95
20	0.1	5	95

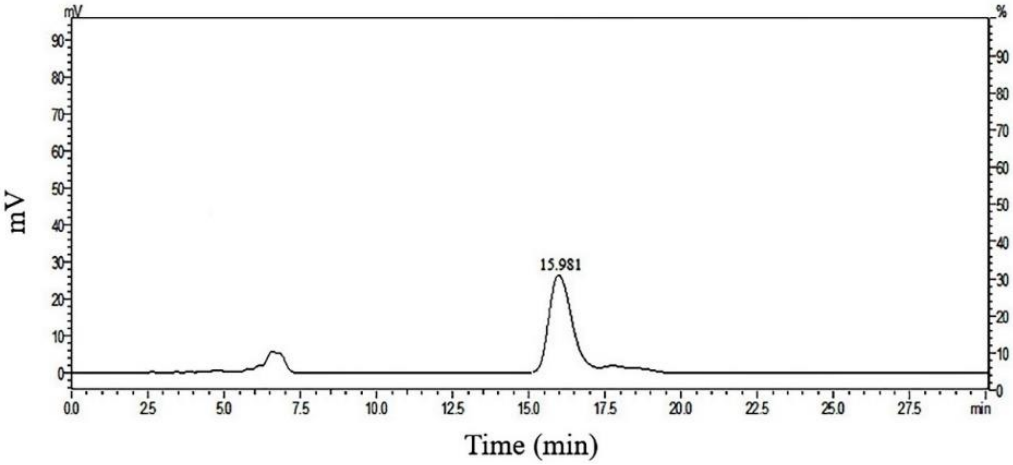

Figure S1. HPLC chromatogram of the purified pigment from silica gel column chromatography.
